# Theoretical and Experimental Study on Vibration Propagation in PMMA Components in Ultrasonic Bonding Process

**DOI:** 10.3390/mi8030092

**Published:** 2017-03-20

**Authors:** Yibo Sun, Feng Wang, Xinhua Yang

**Affiliations:** 1 School of Electric Multiple Unit Application and Maintenance Engineering, Dalian Jiaotong University, Dalian 116028, China; wangfeng@djtu.edu.cn; 2 Dalian Key Laboratory of Welded Structures and Intelligent Manufacturing Technology of Rail Transportation Equipment, Dalian Jiaotong University, Dalian 116028, China; yangxh@djtu.edu.cn

**Keywords:** ultrasonic bonding, micro assembly, vibration propagation, interfacial fusion

## Abstract

Ultrasonic bonding has an increasing application in the micro assembly of polymeric micro-electro mechanical systems (MEMS) with high requirements for fusion precision. In the ultrasonic bonding process, the propagation of ultrasonic vibration in polymer components is related to the interfacial fusion, which can be used as a monitoring parameter to control ultrasonic energy. To study the vibration propagation in viscoelastic polymer components, finite element analysis on the bonding of poly methyl methacrylate (PMMA) micro connector to substrate for microfluidic system is carried out. Curves of propagated vibration amplitude corresponding to interfacial temperatures are obtained. The ultrasonic vibration propagated in PMMA components are measured through experiments. The theoretical and experimental results are contrasted to analyze the change mechanism of vibration propagation related to temperature. Based on the ultrasonic bonding process controlled by the feedback of vibration propagation, interfacial fusions at different vibration propagation states are obtained through experiments. Interfacial fusion behavior is contrasted to the propagated vibration amplitude in theoretical and experimental studies. The relation between vibration propagation and fusion degree is established with the proper parameter range for the obtained high quality bonding.

## 1. Introduction

Thermoplastic polymers are widely used in micro-electro mechanical systems (MEMS) because of their good mechanical properties and easy processing. For example, poly methyl methacrylate (PMMA) and polyether ether ketone (PEEK) are applied to be the base of microfluidic chips and micro pumps [1]. This leads to the need for a precise package of polymeric micro parts in MEMS manufacturing. Nowadays, there are several bonding techniques for the micro assembly of polymeric composites, such as adhesive bonding, thermal bonding, microwave bonding, laser bonding, and ultrasonic bonding.

Ultrasonic bonding is a rapid packaging technology with the advantages of local heating, high efficiency, and no need for pretreatment. It has been applied in MEMS since 2006. Truckenmueller applied plastic ultrasonic bonding in the sealing of microfluidic chips and the assembly of micro pumps [1]. In their research, micro channels made of PMMA were sealed and piezo-driven micro pumps made of PMMA and PEEK were successfully assembled. Kim introduced two ultrasonic bonding systems for metal wire and polymeric MEMS devices [2]. It can be extended to wafer level packaging with low bulk temperature by ultrasonic bonding technique. Ng attempted ultrasonic bonding for the assembly of connectors to microfluidic devices [3]. The PMMA rod connector was bonded with an external diameter of 3 mm, and a 1 mm diameter conduit was bonded on substrate, which was able to withstand a minimum of 6 bars pressure for at least 10 min. In the literature above, the traditional ultrasonic bonding methods for the parts in large scale were adopted with the controlling parameters of time, pressure, electrical energy, or collapse displacement being utilized. The parameters in these modes were directly related to the loading condition of ultrasound, and considered to be open loop control methods.

There are several studies on the improvement of ultrasonic bonding control methods. Mazuzawa studied the information contained in the radiating ultrasound during ultrasonic bonding and proposed that the radiated ultrasound related to the mechanical impedance could be a control parameter [4]. Qiu proposed an ultrasonic method with interposed sheet to impose longitude ultrasound, which is effective for heterogeneous material welds [5]. To improve the control precision of fusion bonding for micro joint, Sun proposed an ultrasonic bonding method based on ultrasonic propagation [6]. This method utilized the vibration propagated in polymer components as a feedback parameter to control the energy, which made the interfacial fusion degree much more precisely controllable.

Ultrasonic has the function of characterizing polymer state. Wang utilized ultrasonic wave to characterize polymer as the energy loss or velocity of ultrasound changes when propagated in varied mediums for melts of Polypropylene (PP), High Density Polyethylene (HDPE), and Polystyrene (PS) [7]. Benatar studied the wave propagation in viscoelastic rods. The Pochhammer frequency equation was simplified for low- and intermediate-loss viscoelastic materials, and corrections for geometric dispersion were formulated for both the phase velocity and attenuation [8]. Lionetto characterized the ultrasonic wave propagated in polymers as a high frequency dynamic mechanical deformation that could be used to monitor the changes of the polymer properties associated with glass transition, crystallization, cross-linking, and other chemical and physical phenomena related to changes in viscoelastic behavior [9].

In ultrasonic bonding, the fusion in the interfacial polymer process is complex. Under ultrasonic load, interfacial temperature rises based on the effects of frictional heating and viscoelastic heating. Simultaneously, the interfacial polymer changes from glassy state to viscoelastic state along with the temperature rising. In this process, ultrasonic vibration propagated in the fusion interface changes, which is a potential monitoring parameter for fusion polymer states. For the ultrasonic bonding process, Benatar proposed a five-part model, including mechanics and vibration of the parts, viscoelastic heating, heat transfer, flow and wetting, and intermolecular diffusion [10,11]. The model was verified by measuring the dynamic mechanical impedance via measurements of both the power and the acceleration on the base. Villegas carried out an experimental analysis of the transformations and heating mechanisms in the ultrasonic bonding process. The relationship between the dissipated power and the displacement of the sonotrode was studied to enable straightforward monitoring of the process through the feedback provided by the ultrasonic welder [12].

There are a few studies regarding the interfacial fusion process in ultrasonic bonding, but little research has focused on the vibration propagation and its relation with interfacial fusion. This paper carries out the theoretical and experimental analysis for the vibration propagation in the ultrasonic bonding process. Finite element analysis is utilized to study the vibration propagation in PMMA components at several temperatures. Test bench with a high frequency dynamic force sensor installed in the anvil is established. The vibration propagated from the ultrasonic horn to the anvil is measured through experiments. Vibration propagation and interfacial fusion behavior in ultrasonic processes are analyzed based on the theoretical and experimental results.

This paper is organized as follows: Section 2 briefly introduces the model, parameters and basic theory of the simulation; theoretical results are discussed in Section 3; and Section 4 illustrates the ultrasonic bonding test bench and the measuring method. Ultrasonic propagation, interfacial fusion, and the relationship between them are discussed in Section 5. Lastly, conclusions are drawn in Section 6.

## 2. Theory

**Figure 1:**
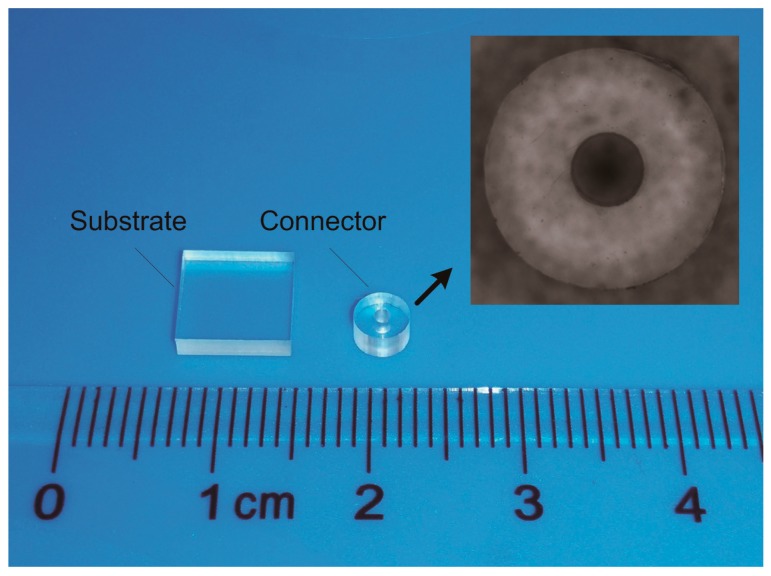
Micro connector and substrate for bonding experiments.

In many microfluidic devices there are micro connectors as the inlet or outlet for fluid supply [3]. To obtain the general law of vibration propagation in the ultrasonic bonding process, a simplified pair of workpieces, shown in Figure 1, is made as the research target for both theoretical and experimental analysis. The external and internal diameters of the connector are 4 and 1 mm, respectively. As one of the amorphous thermoplastic polymers that are commonly used in MEMS, PMMA is used for the workpieces.

**Figure 2:**
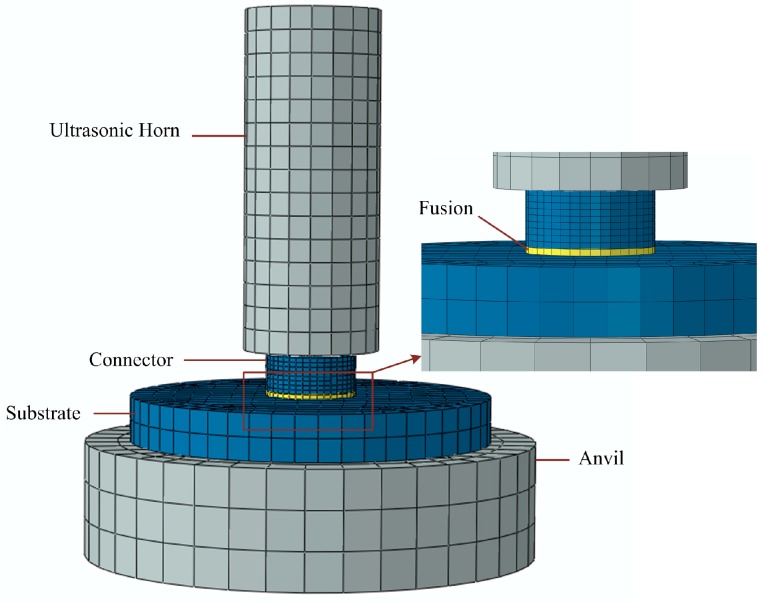
Finite element model corresponding to ultrasonic welding process.

Corresponding to ultrasonic bonding experiments, a finite element analysis model is set up, as shown in Figure 2. Ultrasonic horn and anvil are set to steel as elastic body. Polymer components, including micro connector and substrate, are modeled between horn and anvil, and are set to PMMA. The parameters of the two materials are shown in Table 1.

In the ultrasonic bonding process, heat was generated at the interface and only affected the local region nearby, so that physical state transition only occurred in a small layer of polymer. To simplify this process, a fusion layer in viscoelastic state is attached to the bottom of micro connector by tie contact. In the model, only the mechanical properties of the fusion layer changes with temperature.

The fusion layer in viscoelastic material is the key factor which has the greatest influence on vibration propagation, because the mechanical state clearly changes with rising temperature. The viscoelastic model is utilized to describe the isotropic rate-dependent material behavior for materials in which dissipative losses are primarily caused by “viscous” (internal damping) effects. The shear and volumetric behaviors are assumed to be independent of multiaxial stress states. In the ultrasonic bonding, small strain γ(*t*) is applied to the material. The shear stress τ(*t*) in the viscoelastic material model is defined as:

(1) $$\tau (t)=\int_{0}^{t}G_{R}(t-s)\overset{•}{\gamma }(s)ds$$

**Figure 3:**
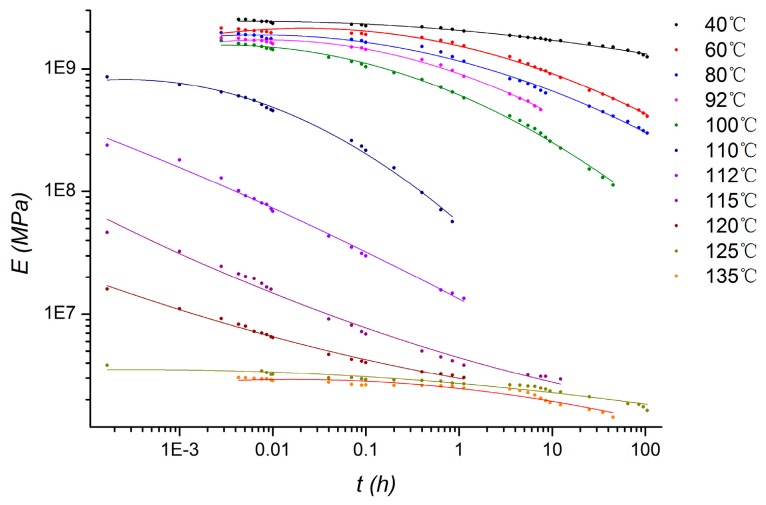
Stress relaxation curves of poly methyl methacrylate (PMMA) at different temperature.

Considering a harmonically varying shear strain γ(*t*) is applied:

(2) $$\gamma (t)=\gamma_{0}\text{ exp}(i\omega t)$$

where γ_0_ is the amplitude, ω is the circular frequency, and *t* is time. The oscillation is assumed to be steady. According to the Boltzmann superposition principle, the solution for the shear stress then has the form

(3) $$\tau (t)=(G_{s}(\omega )+G_{l}(\omega ))\cdot \gamma_{0}\text{ exp}(i\omega t)$$

where *G_s_* and *G_l_* are the shear storage and loss modulus, respectively. These modulus can be expressed in terms of the complex Fourier transform g^*^(ω) of *g*(*t*) in the form

(4) $$g(t)=\frac{G_{R}(t)}{G_{\infty }}-1$$

where *G_∞_* is the long-term shear modulus. Then *G_s_* and *G_l_* can be expressed as

(5) $$G_{s}(\omega )=G_{\infty }(1-\omega \text{Im}(g^{*}))$$

(6) $$G_{l}(\omega )=G_{\infty }(\omega \text{Re}(g^{*}))$$

where Re(g^*^) and Im(g^*^) are the real and imaginary parts of g^*^(ω). The shear relaxation modulus *G_R_*(*t*) of viscoelastic material can be calculated from the stress relaxation curves. The viscoelastic data is derived from the relaxation curves of PMMA at different temperatures in previous work by our research team, as shown in Figure 3 [13].

Three steps simulation based on transient implicit dynamic analysis are set up, corresponding to the pressing, ultrasound loading, and releasing in the ultrasonic bonding process. In the pressing step, pre-tightening force of 10 N is imposed from horn to the polymer components. Then, in the ultrasound loading step, the force is controlled by the displacement, which is consistent with the pressure mechanism in practice. Simultaneously, vibrations of 60 kHz in frequency and 2 μm in amplitude are imposed on the horn, and then propagated in polymer components to the anvil. The vibration load is controlled by displacement, which conformed to the actual load in experiments. In the final step, the horn is moved up with all loads released. As the viscoelastic property of PMMA is corresponding to 11 temperatures, 11 groups of simulation are calculated according to the curves at temperatures of 40 °C, 60 °C, 80 °C, 92 °C, 100 °C, 110 °C, 112 °C, 115 °C, 120 °C, 125 °C, and 135 °C in Figure 3.

In the test bench, high frequency dynamic force sensor is fixed in the anvil to measure the vibration from the bottom of substrate. To make a comparative analysis of theoretical and experimental results, contact force marked as *F_s-a_*, corresponding to the substrate and anvil pair of contact surfaces, is calculated and recorded versus time in the ultrasound loading step.

## 3. Theoretical Results and Discussion

**Figure 4:**
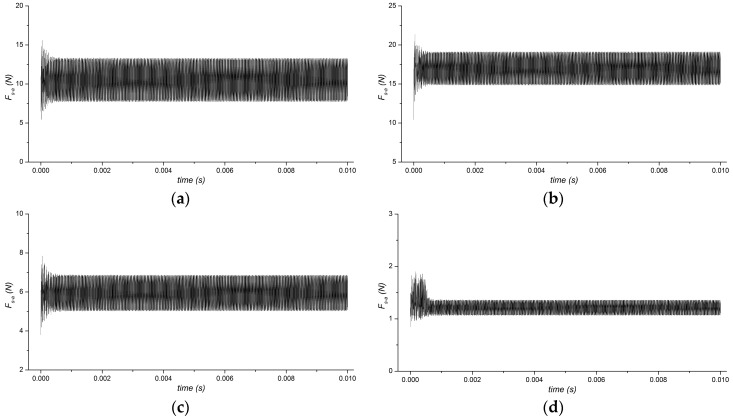
Contact forces in ultrasound loading step at (**a**) 40 °C, (**b**) 110 °C, (**c**) 120 °C, and (**d**) 135 °C.

The contact force of *F_s-a_* between substrate and anvil, which relates to the vibration measured in this experiment, is studied first. The vibration curves, corresponding to time at each temperature, are recorded in the calculation process. Several representative temperatures, including the lowest and highest temperature of 40 °C and 135 °C, and another two of 110 °C and 120 °C which are in the glass transition region, are shown in Figure 4. 

Comparing the four curves, fluctuations occur at all the initial periods when ultrasonic vibration is imposed. Afterwards, the amplitudes of dynamic forces *F_s-a_* tend to be stable. Although the curves have similar tendencies, the values are different. The mean value of *F_s-a_* is about 10 N at 40 °C, increases to about 17 N at 110 °C, decreases to about 6 N at 120 °C, and finally goes down to about 1 N at 135 °C. The amplitude of *F_s-a_* is almost the same at the first three temperature measurements; however, it clearly decreases when temperature rises up to 135 °C. From the results, it is found that temperature in the local layer has great influence on ultrasonic propagation.

**Figure 5:**
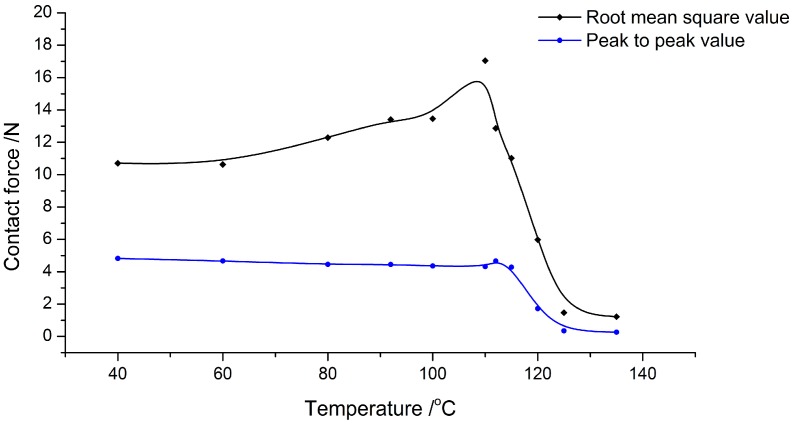
Root mean square value and peak to peak value of *F_s-a_*, corresponding to temperature.

To study the relationship between vibration propagation and temperature, the root mean square value and peak to peak value of *F_s-a_* are calculated corresponding to the curves. The data points are fitted by B-spline to indicate the general variation laws along the temperature, as shown in Figure 5. It is found that the root mean square value increases slowly from 10 N to about 13 N in the range of 40 °C to 100 °C. When the temperature goes up to 110 °C, it increases steeply and reaches the peak at about 17 N. Afterwards, rapid attenuation emerges with the continuous rising of temperature up to 135 °C. In contrast, the peak to peak value is stable in the range from 40 °C to 100 °C. When the temperature goes up over 110 °C, it noticeably attenuates.

The results reflect the physical processes of PMMA with temperature change. PMMA is one type of amorphous polymer with *T_g_* at about 103 °C. Below *T_g_*, it behaves as glass state with little change in the elastic modulus. At temperatures from 40 °C to 100 °C, ordinary elastic deformation occurs under vibration load with nearly no internal energy loss in the viscoelastic polymer structure. As a result, the amplitude of stress in fusion layer is generally stable in this temperature range. Although the elastic modulus is almost the same at temperatures below *T_g_*, the structure will expand with temperature rising. The expansion would introduce thermal stress in the polymer structure, which contributes a static force, but has no effect on the amplitude of dynamic force *F_s-a_*.

When temperature goes up over *T_g_* the mechanical property of PMMA changes, such as the attenuation of elastic modulus, increase of loss factor, and enhancement of creep. During the drastic decrease of elastic modulus, the strain of fusion layer increases under the same load. Thus, the force-propagated vibration steeply decreases within the temperature range from 120 °C to 135 °C. Also, in the glass transition process, energy loss under dynamic load increases as the internal friction is enhanced in viscous body, which is another contribution to the decrease of the peak to peak value of *F_s-a_*.

## 4. Experimental

**Figure 6:**
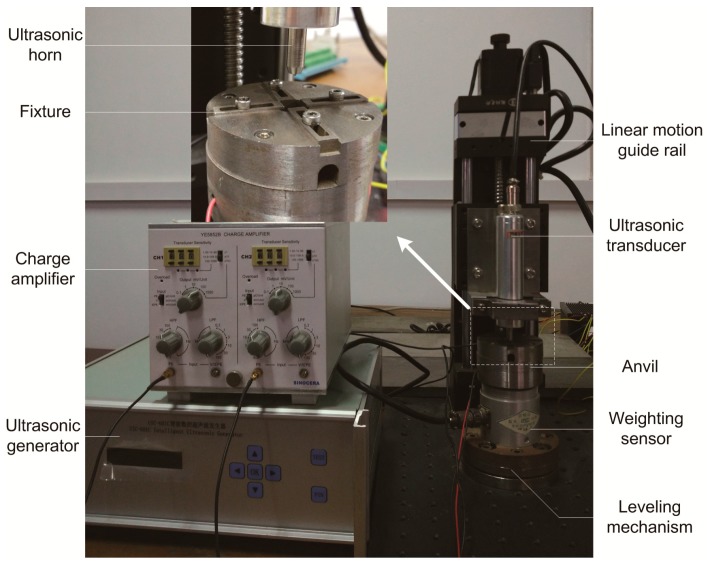
Ultrasonic bonding test bench.

An ultrasonic bonding test bench is established to measure the propagation of ultrasonic vibration, as shown in Figure 6. The ultrasonic system in 60 kHz, including ultrasonic generator, transducer, and horn are chosen to provide ultrasonic vibration. The ultrasonic horn is fixed on a linear motion guide rail and the movement is controlled by stepper motor. In this mode, the force from the horn loaded on the specimen could be controlled more accurately, and the disturbance from pressure float is also avoided. It is a displacement control mode instead of force control mode, which is consistent with the finite element model.

**Figure 7:**
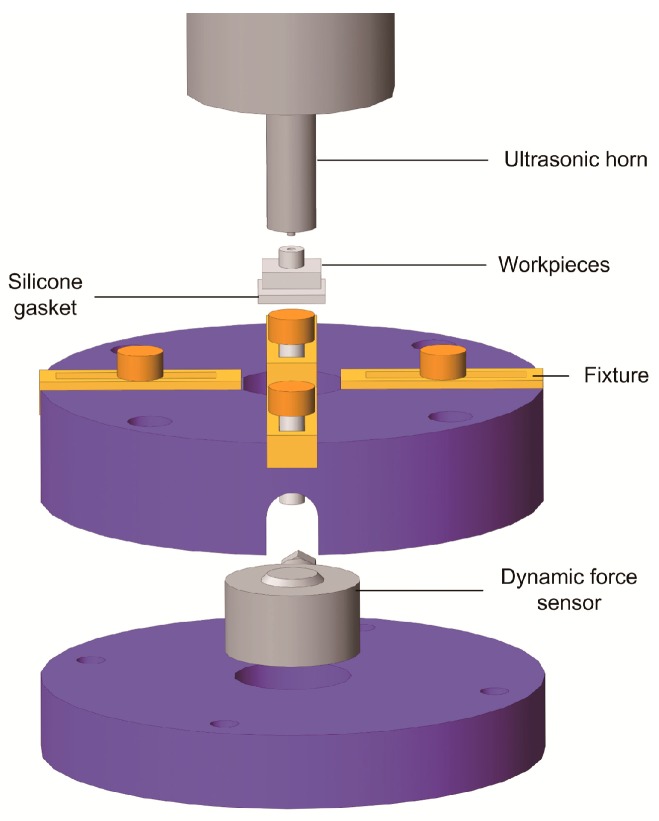
Structure of anvil equipped with dynamic force sensor.

A dynamic force sensor is equipped in the anvil to measure the ultrasonic vibration propagated to the bottom of the polymer substrate. The measuring mechanism is shown in Figure 7. Ultrasonic vibration is loaded from the horn to the polymer workpiece. Silicone gasket is placed between the substrate and the dynamic force sensor to avoid hard impact. As it will not be affected seriously by the interfacial heat through the PMMA substrate, the mechanical property hardly changes in the ultrasonic bonding process, so it does not influence the measured tendency of vibration propagation. The fixture is designed to avoid the horizontal movement of the substrate. Below the dynamic force sensor, an acceleration sensor is installed to measure the acceleration with the purpose of verifying the dynamic force by comparing the waveform. The signals from the two sensors are amplified by charge amplifier and then input into the control system. The anvil is installed above the weighting sensor, which measures the static force.

## 5. Experimental Results and Discussion

### 5.1. Ultrasonic Propagation

**Figure 8:**
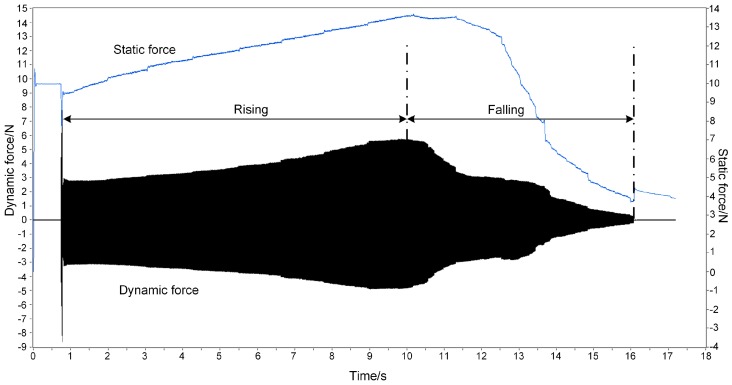
Typical dynamic force and static force in ultrasonic bonding process.

Ultrasonic bonding experiments are set up to measure the propagation of ultrasonic vibration. The vibration propagated from horn to the anvil is measured with parameters of bonding pressure and ultrasonic amplitude set to 10 N and 2 μm, respectively. Since the weighting sensor has low bandwidth, it cannot respond to the high frequency ultrasonic vibration. This static force output is illustrated as the gently-changing blue curve in Figure 8. On the contrary, as the dynamic force sensor has high bandwidth, it ignores the changes in low frequency. This dynamic force output is shown as the black curve with zero mean value in Figure 8.

From the static force curve, there is a violent disturbance at about 0.5 s at the beginning of ultrasonic opening. It then goes up from 10 N, up to peak at around 13.5 N at 10 s with a steady rate. Afterwards, it slowly drops from 10 s to about 12 s, and then sharply from 12.5 s to 16 s. Finally, it falls to about 3.5 N when the ultrasonic load ends.

A similar tendency is found in the dynamic force curve. An impulse appears in the initial ultrasound loading moment at about 0.5 s. The peak to peak value of dynamic force then increases with a steady rate. At about 9 s, it increases from about 6 N in the initial moment, up to about 11 N. Eventually, it decreases from 9 s to 15 s at a fluctuant rate to less than 1 N.

In the ultrasonic bonding process, the interfacial temperature rises, causing the change of vibration propagation. In Figure 8, compared to the theoretical results ignoring the rate at which the temperature rises, there is a similar tendency comparing the root mean square value in Figure 5 and the static force in Figure 8. There are two phases in both curves performed as rising and falling. The theoretical results are in agreement with the experimental ones. It is proven by the experimental results that the rising of static force is caused by the thermal expansion of polymers, whereas the decline is related to the drop of elastic modulus.

The curves from the experimental results are nearly in accordance with the theoretical results; however, there are some differences. For the amplitude of dynamic force, there are two phases in the curve, similar to the static force curve, which are divided into rising and falling phases. The falling phase in Figure 8 accords with the peak to peak value in Figure 5, while the rising dose not. In the theoretical model, the ultrasonic load is invariant at every temperature and also irrelevant to the static load. However, in these experiments, the output of the ultrasonic transducer is affected by the load on the horn.

**Figure 9:**
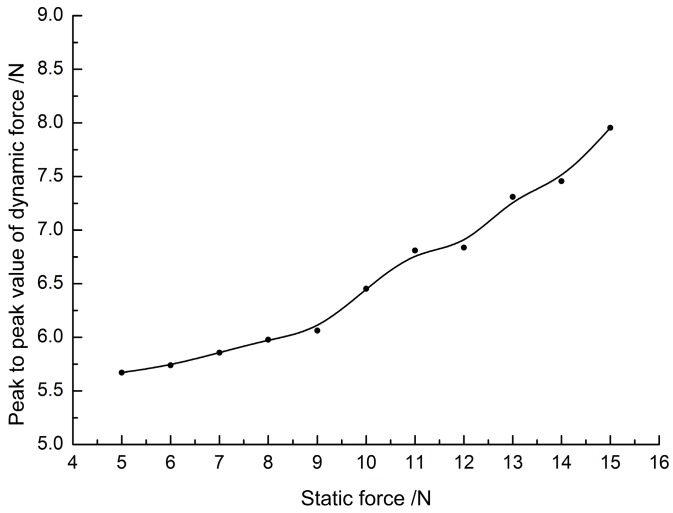
Peak to peak value of measured dynamic force at different preload.

To find out why the vibration amplitude rises in Figure 8, the relationship between output dynamic force from the ultrasonic horn and the preload are tested. Ultrasonic horn is imposed on the anvil without polymer specimens to measure the dynamic force directly. The peak-to-peak values corresponding to preload values are fitted by B-spline to indicate the general tendency, as shown in Figure 9. It is found that the output force increases with preload. This is due to the ultrasonic energy being controlled by amplitude in the test bench, and the horn providing larger value to maintain the amplitude of ultrasonic output when the preload increases. Then, returning to the curves in Figure 8, the rise of static force in the first period causes the enhancement of ultrasonic output, and eventually causes the amplitude increase of dynamic force.

The amplitude attenuation of dynamic force is also caused by the glass transition of local polymer. The attenuation of elastic modulus and the enhancement of energy loss are the main contributions. A key point is that the time point for the turning from rising to falling is hysteretic compared to the dynamic force curve. The reason is that the weighting sensor needs time to respond to the load change. This also leads to a difference of attenuation tendency in the falling phase. Therefore, it is preferable to monitor the process by dynamic force, which has a high-speed response.

### 5.2. Interfacial Fusion

To study the relation between the state of polymer and ultrasonic propagation, ultrasonic bonding experiments controlled by vibration propagation are carried out. The process is as follows: first, a target change rate of the dynamic force amplitude is set as a parameter to turn off ultrasonic energy. In the bonding process, the initial amplitude of vibration in a stable state after the impulse, as shown in Figure 9, is recorded as the reference amplitude. Afterwards, the real-time amplitude of dynamic force is calculated to the ratio operation with the initial one. The ultrasonic generator is shut down at the time that the real-time change rate reaches the setting value. As a result, the interfacial polymer fusion degree can be obtained at any vibration propagation state.

**Figure 10:**
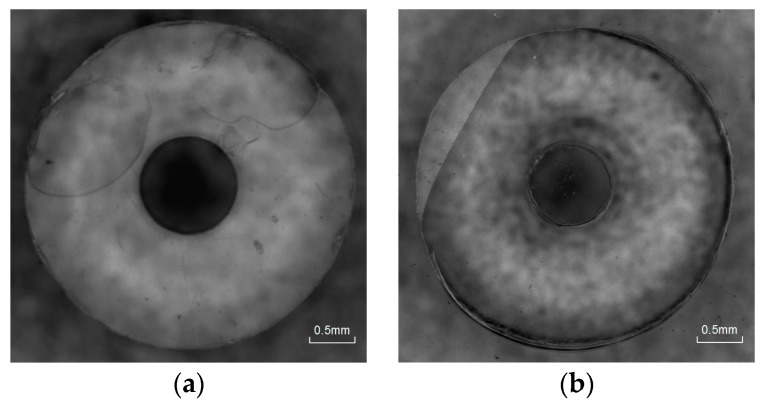

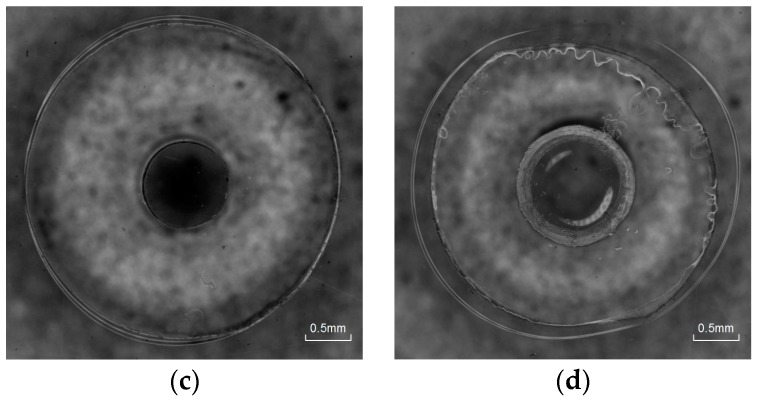
Interfacial Fusion at change rate of (**a**) 130%, (**b**) 100%, (**c**) 60%, and (**d**) 10%.

Four representative parameters of vibration propagation changing rate (130%, 100%, 60%, and 10%) are presented in Figure 10.

At the change rate of 130%, fusion bonding cannot be achieved; only a partial area near the edge melted, whereas most other regions remained undisturbed. In the initial period, heat initiates in some regions with close contact by high frequency repeated impact under ultrasonic load, and then spreads around gradually. Corresponding to the theoretical and experimental dynamic force curves in the rising phase, the local temperature is close to *T_g_*. In this state, the polymer molecular chain is not fully activated. The conversion of vibration energy loss in viscoelastic body to heat cannot contribute much. The locations of the melting regions are random, which depend on the friction of two surfaces.

At the change rate of 100%, most areas achieved fusion bonding, except for a small part on the edge. With the continuous attenuation to 60%, fusion spread all over the interface. According to the discussion above, the amplitude of dynamic force rises as the ultrasonic output increases with the static load. When it falls back to the value that is equal to the initial value, it is equivalent to the initial falling phase in the theoretical results in Figure 5. Thus, the temperature rises over *T_g_* in this case. The polymer molecular chain is activated in the glass transition region. In this phase, the heat is mainly generated by the energy dissipation in viscoelastic body, which is more efficient than frictional heat. Thus, the attenuation of vibration propagation efficiency is rapid. Melting also spreads more quickly as the mobility of molecular chains is enhanced at the temperature that is higher than *T_g_*.

When the change rate is decreased to 10%, overflow becomes more obvious and bubbles begin to generate on the edge of inner circle. In this case, a higher interfacial temperature is achieved. Polymer is in viscous flow state with fluid characteristics enhancing gradually. The ultrasonic cavitation effect functions with bubbles being generated. Some materials are also squeezed out of the edge.

## 6. Conclusions

The changing of vibration propagation in the ultrasonic bonding process is studied by theoretical and experimental analysis in this paper. From the theoretical results, with the rising of temperature, the root mean square value of the dynamic force acting on the anvil increases below *T_g_*, which is caused by expansion; and decreases dramatically over *T_g_*, which is caused by attenuation of elastic modulus. For peak-to-peak value, it is relatively stable below *T_g_* and also drops dramatically over *T_g_*, which is contributed to by the increase of energy loss under dynamic load as internal friction is enhanced in the viscous body. Combining the theoretical and experimental results, the change tendency of vibration propagation is almost consistent except for the peak to peak value of dynamic force. It is verified by experiment that this is caused by the output increase of the ultrasonic system under larger preload.

The relation between the state of polymers and ultrasonic propagation is also studied. Ultrasonic bonding experiments controlled by vibration propagation are carried out. Based on the dynamic force curves at the change rate of 130%, only a partial area near the edge melted, which is related to the interfacial temperature close to *T_g_*. With the decrease of amplitude, fusion spreads gradually and the whole interface fusion is achieved at a change rate of 60%. This process is related to the glass transition region. With the amplitude change rate down to 10%, overflow becomes more obvious and bubbles begin to generate as the ultrasonic cavitation effect in viscous flow polymer.

## Figures and Tables

**Table 1:** Material parameters for steel and poly methyl methacrylate (PMMA) parts.

Materials	Young’s Modulus (Pa)	Poisson’s Ratio	Density (kg/m^3^)
Steel	2 × 10^11^	0.27	7890
PMMA	3 × 10^9^	0.37	1190
